# Sexual-biased gene expression of olfactory-related genes in the antennae of *Conogethes pinicolalis* (Lepidoptera: Crambidae)

**DOI:** 10.1186/s12864-020-6648-3

**Published:** 2020-03-19

**Authors:** Dapeng Jing, Tiantao Zhang, Shuxiong Bai, Kanglai He, Sivaprasath Prabu, Junbo Luan, Zhenying Wang

**Affiliations:** 1 State Key Laboratory for Biology of Plant Diseases and Insect Pests, Institute of Plant Protection, Chinese Academy of Agricultural Sciences, Beijing, 100193 China; 2 College of Plant Protection, Shenyang Agricultural University, Shenyang, 110161 China

**Keywords:** *Conogethes pinicolalis*, *Conogethes punctiferalis*, Yellow peach moth, Transcriptomics, OBP, GOBP, PBP, RNA-Seq, Transcriptome

## Abstract

**Background:**

*Conogethes pinicolalis* (Lepidoptera: Crambidae), is similar to *Conogethes punctiferalis* (yellow peach moth) and its host plant is gymnosperms, especially for masson pine. So far, less literature was reported on this pest. In the present study, we sequenced and characterized the antennal transcriptomes of male and female *C. pinicolalis* for the first time.

**Results:**

Totally, 26 odorant-binding protein (OBP) genes, 19 chemosensory protein (CSP) genes, 55 odorant receptor (OR) genes and 20 ionotropic receptor (IR) genes were identified from the *C. pinicolalis* antennae transcriptome and amino sequences were annotated against homologs of *C. punctiferalis*. The neighbor-joining tree indicated that the amino acid sequence of olfactory related genes is highly homologous with *C. punctiferalis*. Furthermore, the reference genes were selected, and we recommended the phosphate dehydrogenase gene (GAPDH) or ribosomal protein 49 gene (RP49) to verify the target gene expression during larval development stages and RP49 or ribosomal protein L13 gene (RPL13) for adult tissues.

**Conclusions:**

Our study provides a starting point on the molecular level characterization between *C. pinicolalis* and *C. punctiferalis*, which might be supportive for pest management studies in future.

## Background

Olfaction system plays a key role in insects, which includes kin recognition, mediating foraging, aggregation, toxic compound avoidance and oviposition behaviors. However, the olfaction is a complex network that contains odorant-binding proteins (OBP), odorant receptors (OR), chemosensory proteins (CSP), sensory neuron membrane proteins (SNMPs), ionotropic receptors (IR) and odorant degrading enzymes (ODEs). They form a functional network with each other in detecting different odorants types, thus complete the odorants recognition process [1, 2]. In Lepidoptera, OBPs are composed of pheromone-binding proteins (PBPs), general odorant-binding proteins (GOBPs) and antennal binding proteins (ABPs), and they combined to detect a wide range of odors and transport hydrophobic odorants to the ORs or IRs [3]. The functions of CSPs are also similar to OBPs, localized in the lymph of trochoid sensilla [4]. IRs or ORs are localized on the dendrite of the chemosensory neuron, which can transform the chemical signals from OBPs or CSPs into an electric signal and transmit to the brain [5, 6]. The SNMPs and ODEs are regarded to trigger ligand delivery to the receptor and terminate the signal stimulation, respectively [6].

*Conogethes pinicolalis* (Lepidoptera: Crambidae), is a sibling species of *Conogethes punctiferalis* (Lepidoptera: Crambidae). Morphological features of *C. pinicolalis* egg, larva, pupa and adult resemble those of *C. punctiferalis* and it is considered as same species. In 1963, Koizumi firstly identified the *C. pinicolalis* as an another type of yellow peach moth and classified as pinaceae-feeding type (PFT) [7]. Later, Honda and Mitsuhashi identified and distinguished the difference between these pests in the adults, larvae and pupal stages [8]; Konno et al. reported that they were different species from their response to different spectra of host-plant constituents [9]; In 2006, the pinaceae-feeding type was named as *C. pinicolalis* [10]. Though these studies have provided important information regarding the identification of species, it is not entirely reliable because these insect groups were undergoing speciation, genomic changes, or evolving into new taxon [11]. Therefore, for its high reliability, molecular characterization technique can serve as a complementary method for further analysis. Especially, DNA sequencing and mitochondrial DNA (mtDNA) have been successfully used to deal with the species uncertainty in morphological taxonomy [12–14]. For example, Shashank integration of conventional taxonomy, DNA bar code and others methods successfully confirmed the difference in populations of *Conogethes* which reared on castor and cardamom in India [11]. Furthermore, Wang et al. used mitochondrial DNA sequencing technique to verify *C. pinicolalis* and *C. punctiferalis* were significantly different species [15].

*C. pinicolalis* is a typical oligophagous pest that can only feed on *Pinus massoniana* (masson pine) and few pine trees. However, as a sibling species, *C. punctiferalis,* is a polyphagous pest that can infest hundreds of plants [9, 16]. High-throughput sequencing technology can provide us with a lot of data and it has greatly promoted the research on entomology [17, 18]. In this study, we analyzed the difference of male and female antennae transcriptome and identified the olfactory genes from Gene Ontology (GO) annotation as well as sets of putative OBPs, CSPs, ORs and IRs in *C. pinicolalis*. Furthermore, we compared the difference of the genes with *C. punctiferalis*. These results provide basically data for the study of *C. pinicolalis* olfactory genes, also may help to better understand the genetic evolution between these two sibling species.

## Results

### Overall sequence analysis

A total of 78,199,136 and 75,969,652 raw reads were obtained from male and female antennae, respectively. We obtained 77,254,390 and 74,994,240 clean reads from male and female antennae after trimming adapter sequences, eliminating low-quality reads, and N represented sequences. A total of 98,214 unigenes were obtained with an average length of 815 bp and with a N50 of 2968 (Table 1). The raw reads of the *C. pinicolalis* are available from the SRA database (accession number: SRX5250688, SRX5250689, SRX5250690, SRX5250691, SRX5250692 and SRX5250693).

**Table 1:** Summary of assembled contigs and unigenes

Type (bp)	Contigs	Unigenes
Total number	121,650	98,214
Total length	160,640,609	154,441,888
Min length	201	201
Mean length	568	815
Maximum length	25,856	25,856
N50	2825	2968
N90	467	612

### Functional annotation of the *C. pinicolalis* antennal unigenes

In total, 98,214 unigenes were successfully annotated in all databases (Table 2), including 47,089 (47.94%) unigenes matched to known proteins and 33,852 unigenes (34.46%) in the Swiss-Prot database. GO analysis was used to classify the biological process, molecular function and cellular components (Additional file 1: Figure S1A). Under the molecular function category, the genes expressed in the antennae were mostly related to binding, catalytic activity and transporter activity (Additional file 1: Figure S1B). From the Kyoto Encyclopedia of Genes and Genomes (KEGG) annotation, 10,298 unigenes were classified into five groups, cellular processes, environmental information processing, genetic information processing, metabolism and organismal systems (Additional file 1: Figure S1C).

**Table 2:** Summary of annotations of unigenes

Type (bp)	Number of Unigenes	Percentage (%)
Annotated in NR	47,089	47.94
Annotated in NT	31,124	31.68
Annotated in KO	18,774	19.11
Annotated in SwissProt	33,852	34.46
Annotated in PFAM	37,710	38.39
Annotated in GO	37,882	38.57
Annotated in KOG	19,474	19.82
Annotated in all Databases	8967	9.13
Annotated in at least one Database	59,764	60.85
Total Unigenes	98,214	100

### Olfactory-related genes in the *C. pinicolalis* antennae

**Fig. 1:**
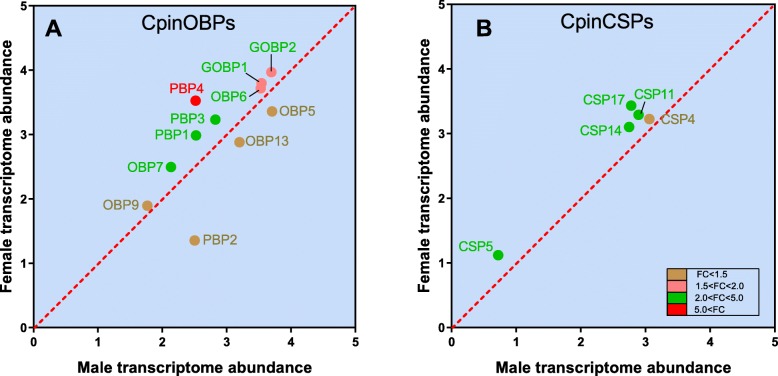
Scatter plots showing the differential regulation of OBP and CSP genes in male and female *C. pinicolalis* antennae. Transcripts that exhibit significant differences in abundance (*P* < 0.05), are color-coded according to their weighted fold change (FC). The expression levels are shown as the mean Log10 (TPM + 1) for all of the three biological replicates for both sexes

Totally, 26 OBP genes, 19 CSP genes, 55 OR genes and 20 IR genes were identified from the *C. pinicolalis* antennae (Additional file 2: Table S1). Among the identified OBP genes, we found 4 PBP, 2 GOBP and 20 other kinds of OBP genes. Furthermore, OBP and CSP genes are detected in male and female antennae and showed the significant differences in genes abundance (*P* < 0.05) (Fig. 1). Interestingly, PBP2, OBP13 and OBP15 are male biased expression, whereas the other PBPs (PBP1, PBP3 and PBP4), as well as GOBPs (GOBP1 and GOBP2) are female bias expression. Furthermore, two of the other OBPs (OBP7 and OBP9) remained female biased expression (Fig. 1a). CSP genes (CSP4, CSP5, CSP14, CSP11 and CSP17) showed female biased expression and significantly different from the male (Fig. 1b), Other insignificantly expressed genes were shown in Additional file 2: Table S1.

**Fig. 2:**
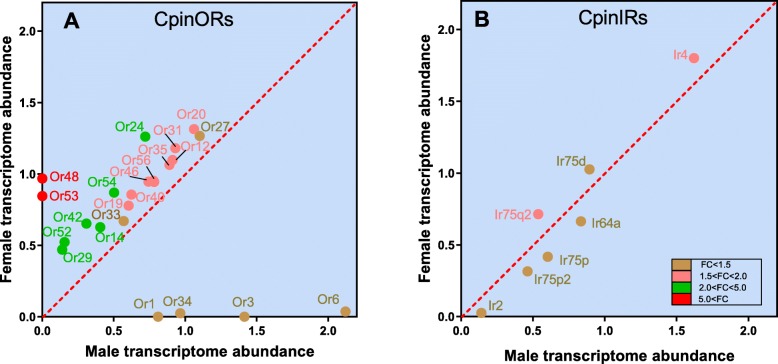
Scatter plots showing the differential regulation of OBP and CSP genes in male and female *C. pinicolalis* antennae. Transcripts that exhibit significant differences in abundance (*P* < 0.05), are color-coded according to their weighted fold change (FC). The expression levels are shown as the mean Log10 (TPM + 1) for all of the three biological replicates for both sexes

In OR gene sets, 7 pheromones receptors (PRs) and 47 other ORs were identified in male and female antennae. Three PR genes (OR1, OR3 and OR6), as well as OR34, showed significantly higher expression in male antennae. However, a large number of ORs (about 18 genes) were significantly higher expression in female antennae. Especially the OR48 and OR53, are highly expressed in female antennae with differential fold change (FC) > 5. Six ORs with 2.0 < FC < 5.0 (*P* < 0.05) and eight ORs with 1.5 < FC < 2.0 (*P* < 0.05) (Fig. 2a). Three IR genes (IR75p2, IR75d and IR4) showed female biased expression (*p* < 0.05) and other four genes (IR2, IR75p2, IR75p, and IR64a) were male biased expression (*p* < 0.05) (Fig. 2b).

Significantly expressed genes were confirmed by quantitative real-time PCR (RT-qPCR) (Additional file 1: Figure S2). Expressions of female biased genes from class OBP (PBP1, PBP3, PBP4, GOBP1, GOBP2, OBP6, OBP7 and OBP9) were enormously consistent with the transcripts per kilobase million (TMP) values.. The same results were obtained in the expression of CSPs, ORs and IRs (Additional file 1: Figure S2).

### Phylogenetic analysis

**Fig. 3:**
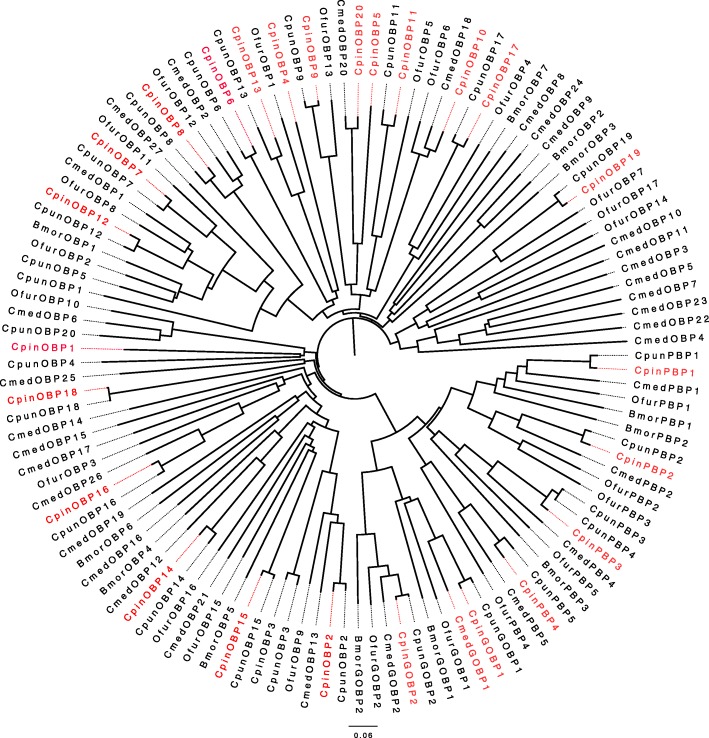
Phylogenetic relationship of olfactory-related gene from *C. pinicolalis* and other insects. Red font represents the genes from *C. pinicolalis*; Cpun, Ofur, Bmor and Cmed are the abbreviation of *C. punctiferalis*, *O. furnacalis, B. mori* and *Cnaphalocrocis medinalis*, respectively

Phylogenetic trees were constructed by using 95 OBPs, 157 ORs, 89 CSPs and 59 IRs from different species of Lepidoptera (Fig. 3; Additional file 1: Figure S3). The GOBP/PBP genes sequences include six subgroups (GOBP1 and 2, PBP1–4) formed a conserved order (Fig. 3). Furthermore, OBPs, CSPs, ORs and IRs showed a very close relationship with *C. punctiferlis,* only a few CSPs and IRs clustered with other insects (Fig. 3; Additional file 1: Figure S3). Most of the olfactory related genes showed more than 90% identity. Moreover, 4 OBP, 5 OR, 2 IR and 2 CSP genes had 99% sequence similarity with the *C. punctiferlis* (Table 3). ORs and IRs genes indicated the *Ostrinia furnacalis* is the next close neighbor in the same clade. On the other hand, OBPs and CSPs genes showed *Cnaphalocrocis medinalisin* in the same clade as a close neighbor after C. *punctiferlis.* Olfactory-related genes in *Bombyx mori* showed gene divergence when compared with these two sibling species.

**Table 3:** Percentage identity of OBP, OR, IR and CSP gene family in *C. pinicolalis with the sibling C. punctiferalis*

Gene family	Gene names	***C. pinicolalis access No.***	***C. punctiferalis access No.***	Score	E-value	% Identity	Gene family	Gene names	***C. pinicolalis access No.***	***C. punctiferalis access No.***	Score	E-value	% Identity
**Odorant-binding proteins**	OBP2	MK458342	KF026055	306	3e-102	97	**Odorant receptors**	OR27	MK458386	KX084477	740	0	99
	OBP3	MK458343	KF026056	210	5e −67	96		OR28	MK458387	KX084478	586	0	94
	OBP4	MK458344	KP985222	278	2e-91	74		OR29	MK458388	KX084479	734	0	88
	OBP5	MK458345	KP985223	180	6e-94	99		OR31	MK458390	KX084481	564	0	83
	OBP6	MK458346	KP985224	249	2e-79	96		OR32	MK458391	KX084482	712	0	93
	OBP7	MK458347	ALC76547	288	2e-95	97		OR33	MK458392	KX084483	774	0	99
	OBP8	MK458348	KP985226	193	1e-94	95		OR34	MK458393	KX084484	444	1e-153	63
	OBP9	MK458349	KY130463	330	2e-112	98		OR35	MK458394	KX084485	882	0	99
	OBP10	MK458350	KY130464	251	1e-82	99		OR36	MK458395	KX084486	409	3e-98	93
	OBP11	MK458351	KY130465	280	4e-89	94		OR37	MK458396	KX084487	735	0	96
	OBP12	MK458352	KY130466	221	2e-50	98		OR38	MK458397	KX084488	657	0	78
	OBP13	MK458353	KY130467	124	1e-34	88		OR41	MK458400	KX084491	644	0	93
	OBP14	MK458354	KY130469	271	2e-34	95		OR42	MK458401	KX084492	686	0	92
	OBP15	MK458355	KY130470	297	1e-97	98		OR43	MK458402	KX084493	581	0	96
	OBP16	MK458356	KY130472	226	3e-72	97		OR44	MK458403	KX084494	684	0	86
	OBP17	MK458357	KY130473	307	3e-104	98		OR45	MK458404	KX084495	508	3e-175	63
	OBP18	MK458358	KY130474	353	5e-115	99		OR47	MK458406	KX084497	299	4e-100	99
	OBP19	MK458359	KY130475	252	1e-82	97		OR48	MK458407	KX084498	437	1e-148	79
	GOBP1	MK458335	KY130468	297	3e-100	95		OR49	MK458408	KX084499	114	3e-23	91
	GOBP2	MK458336	KT983812	191	4e-57	99		OR50	MK458409	KX084500	800	0	94
	PBP1	MK458337	MH006604	192	2e-59	97		OR51	MK458410	KX084501	647	0	93
	PBP2	MK458338	KP985228	190	5e-33	95		OR52	MK458411	KX084502	728	0	91
	PBP3	MK458339	KP985229	338	1e-100	95		OR53	MK458412	KX084503	691	0	90
	PBP4	MK458340	KP985227	329	3e-106	93		OR54	MK458413	KX084504	853	0	92
**Odorant receptors**	OR1	MK458361	KX084452	890	0	95		OR55	MK458414	KX084505	839	0	94
	OR2	MK458362	KX084453	952	0	99		OR56	MK458415	KX084506	690	0	89
	OR3	MK458363	KX084454	641	0	94	**ionotropic receptors**	IR3	MK458418	KX084511	1299	0	99
	OR4	MK458364	KX084455	868	0	92		IR4	MK458419	KX084512	1057	0	98
	OR5	MK458365	KX084456	758	0	95		IR5	MK458420	KX084513	1484	0	81
	OR6	MK458366	KX084457	805	0	95		IR6	MK458421	KX084514	1348	0	81
	OR7	MK458367	KX084458	555	0	90		IR7	MK458422	KX084515	1089	0	97
	OR8	MK458368	KX084459	339	3e-110	77		IR25a	MK458424	KX094508	1797	0	99
	OR10	MK458369	KX084461	656	5e-165	87	**Chemosensory proteins**	CSP1	MK574125	KF026049	154	1e-41	96
	OR11	MK458370	KX084462	683	0	97		CSP2	MK574126	KF026050	259	8e-78	96
	OR12	MK458371	KX084463	664	0	93		CSP3	MK574127	KY130477	191	1e-60	90
	OR13	MK458372	KX084464	752	0	96		CSP4	MK574128	KF026057	226	5e-69	96
	OR14	MK458373	KX084465	798	0	97		CSP5	MK574129	KF026058	246	1e-78	98
	OR15	MK458374	KX084466	673	0	90		CSP6	MK574130	KF026051	228	1e-67	97
	OR16	MK458375	KX084467	794	0	91		CSP7	MK574131	KF026052	201	1e-59	97
	OR17	MK458376	KX084468	790	0	98		CSP8	MK574132	KF026053	172	3e-53	99
	OR18	MK458377	KX084469	786	0	95		CSP9	MK574133	KY130480	241	5e-78	96
	OR19	MK458378	KX084470	726	0	89		CSP10	MK574134	KY130479	197	8e-71	99
	OR20	MK458379	KX084471	729	0	96		CSP11	MK574135	KY130480	219	2e-59	96
	OR21	MK458380	KX084472	536	3e-178	77		CSP13	MK574137	KY130482	206	2e-64	88
	OR23	MK458382	KX084473	730	0	93		CSP14	MK574138	KY130483	228	3e-71	92
	OR24	MK458383	KX084474	897	0	98		CSP15	MK574139	KY130484	237	4e-76	94
	OR25	MK458384	KX084475	833	0	93							

### Reference genes selection

**Fig. 4:**
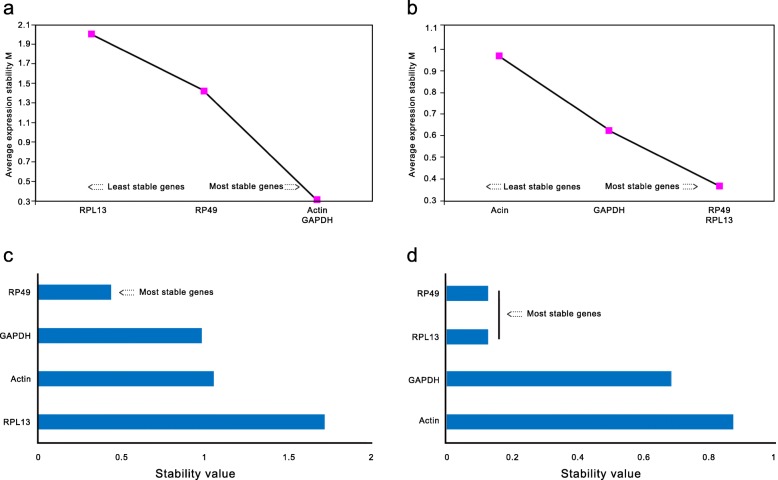
Stability analysis of candidate reference genes in different developmental stages (**a, c**) and different adult tissues (**b, d**) of *C. pinicolalis*

The gene stability results obtained from both the software seems to be similar (Fig. 4). In the adult tissues (antanna, head, throax, abdomen, leg and wings) ribosomal protein 49 gene (RP49) and ribosomal protein L13 gene (RPL13) showed more stability than GADPH gene, and Actin gene was unstable (Fig. 4b and d). However, RPL13 performed unstable in different development stages of the *C. pinicolalis*. The results of GeNorm software showed that Actin and phosphate dehydrogenase gene (GAPDH) are the most stable gene (Fig. 2a); while NormFinder software considered RP49 to be the most stable gene (Fig. 4b).

## Discussion

The application of next-generation sequencing technology in the field of entomology has greatly promoted the efficiency and quantity of gene annotation [19]. Meantime, a lot of antennal transcriptomes olfactory-related genes were identified [20–22]. In this research, we identified 26 OBP genes, 19 CSP genes, 55 OR genes and 20 IR genes from the *C. pinicolalis* antennal transcriptome, these genes have been reported for the first time in this species. *C. pinicolalis* is a sibling species of *C. punctiferlis*, and had ever been recognized as the same species [10]. In *C. punctiferlis*, totally 25 OBPs, 15 CSPs, 62 ORs and 10 IRs were identified from antennae transcriptome [23], and the numbers of OBPs, CSPs and ORs are similar with *C. pinicolalis*, whereas more IRs were identified from the *C. pinicolalis* antennal transcriptome dataset, this may depend on the depth of the sequencing. The sequence similarity of olfactory-related genes was analyzed and shown in the evolution tree (Fig. 3, Table 3), OBP, CSP, OR and IR genes sequences showed high similarity with *C. punctiferlis.* Most of the identities are more than 90%. 4 OBP, 5 OR, 2 IR and 2 CSP genes had 99% sequence similarity with the *C. punctiferlis* (Table 3). These two pests were first identified by Koizumi et al. [7] and classified into pinaceae-feeding type (PFT) and fruit-feeding type (FFT) based on their feeding habits and morphological characters. They were later named as *C. pinicolalis* and *C. punctiferalis* [10]. Further investigation revealed their behaviors, morphologies, and feeding patterns, and indicated reproductive isolation between these two types [9, 16, 18]. Wang et al. have shown that the *C. pinicolalis* was different from that of *C. punciferalis* through mitochondrial cytochrome c oxidase subunits I, II and cytochrome *b* gene sequences [15]. The phylogenetic tree also revealed an evolutionary relationship with other Lepidopteran species. The GOBP/PBP genes sequences include six subgroups (GOBP1 and 2, PBP1–4) formed a conserved order (Fig. 3). ORs and IRs genes indicated the *Ostrinia furnacalis* is also the close neighbor in the same clade (Additional file 1: Figure S3). On the other hand, OBPs and CSPs genes showed *Cnaphalocrocis medinalisin* in the same clade as a close neighbor after *C. punctiferlis.* Olfactory-related genes in *Bombyx mori* showed gene divergence when compared with these two sibling species.

Menken et al. [24] suggested the two major transitions in the evolution of larval (Lepidoptera) feeding, switching from litter-feeding to herbivory. Larvae feeding on leaf-litter from a single dominant tree species would have been the main precursor for evolving from litter-feeding to leaf-mining type. In the course of evolution, leaf-mining type gained the new type of enzymatic system to digest the nutritious freshly fallen leaves. Once this evolved niche had been acquired the ability of leaf-mining and with the special digestive system could apparently exploit the diversity more and larval feeding mode had evolved in searching of new host-plants [25]. Insects olfaction system allows them to recognize and track the volatile cues from host-plant, mating and evade from their predators. The polyphagous insects significantly adapted to recognize, digest and detoxify a large variety of host-plants. Polyphagous insects must handle the defensive toxic molecules (secondary metabolites) produced by the host-plant. Genes from the moth pheromone glands could have evolved and altered the normal fatty acid metabolism [26]. In a previous study, experiments proved the major change in the pheromone blend in various moth species, the existence of different desaturase from mRNA in the moth pheromone gland [27]. In *Spodoptera frugiperda*, due to tandem duplications within a single region of the genome 10 OBP genes expansion was observed when compared with *B. mori*. In the same study, the author showed a difference in IRs gene count between the strains, *S. frugiperda* corn strain had 42 IRs and rice strain had 43 IRs [28]. Similarly, in our study *C. pinicolalis* had 10 more IRs when compared with *C. punctiferlis*. Evidently, the selection of host plant is also a reason that leads to gene duplications, insertions or deletions when there is a need to adapt to an environment.

As in other insects [29–31] OBPs and CSPs were detected in the antennae of both male and female (Additional file 2: Table S1). Among these genes, many of them were sexual biased genes (Fig. 1). PBPs were widely thought to be sex pheromone binding function, normally insects have 3–5 PBP genes. Previous studies suggested that at least one PBP family isoform could well interact with the sex pheromones [32–34]. In our analysis, PBP2 showed significantly male biased expression, and PBP1, PBP3 and PBP4 showed significantly female biased expression. In male moth, the main assignment is to trail the sex pheromones to find a female moth for mating. We speculated the PBP2 might play a critical role in pheromone binding. Females are often selective in seeking a healthy counterpart for mating. GOBP1 and GOBP2 genes, as well as OBP6, OBP7 and OBP9, were also highly expressed in female, this may play some important roles and need for further study. GOBPs are proposed to detect host plants volatiles, food and oviposition sites and PBPs play a key role in detecting sex pheromones [35–37]. However, some studies have demonstrated that GOBPs can interact with sex pheromones and possibly responsible for conducting the function [38]. Our another study have showed that PBP2 and GOBP1 genes may play similar roles in detecting and transporting sex pheromones and host plant volatiles in *C. pinicolalis* [39]. There are also evolutionary evidence that GOBPs may evolved from PBP by gene duplication, PBP and GOBP2 in *Manduca sexta* show close relationship and play an important role in coordinated olfactory behaviors [40, 41]. Although the transcriptome of *C. pinicolalis* and *C. punctiferlis* possess higher similarity, the *C. pinicolalis* adult rely on fresh masson pine branches for laying eggs, which the case is very different in *C. punctiferlis* adult, they have a wide variety of host plants selection. Therefore, both GOBPs and PBPs from *C. pinicolalis* and *C. punctiferlis* might have a greater interest in future research.

CSPs were found in insect contact and sensilla olfactory, but other members exhibited peculiar functions. In *Apis mellifera*, CSPs have been reported to be involved in larval growth and brood pheromone transportation [42, 43]. In a cockroach *Blatta germanica*, a CSP is involved in leg regeneration [44]. CSPs binding affinity towards volatile compounds was similar to that of OBPs [45]. In *C. pinicolalis* antennae transcriptome, we totally identified 19 putative CSPs, and found the transcript per kilobase million (TPM) values of five CSPs (CSP4, CSP5, CSP11, CSP14, and CSP17) were significantly higher in female antennae (Fig. 1b). MsepCSP8 of *Mythimna separate* was specially expressed in female antennae and showed less sensitive to plant volatiles after RNAi [46]. Also in *Locusta migratoria*, nearly 17 CSPs abundantly expressed in the female reproductive organs [47]. Higher numbers of CSPs in female antennae provide a valuable understanding that CSPs may play an important role in female moths, particularly when it comes to tracking the volatile cues from host-plants and oviposite.

Totally there were 55 OR genes identified from male and female antennal transcriptome dataset, among them 22 ORs showed a significant difference in TPM ratio (Additional file 2: Table S1). In Lepidoptera, OR1 and OR3–8 were identified as pheromone receptors (PR). Our result obviously showed OR1, OR3 and OR6 were specially expressed in male antennae, this may suggest OR1, OR3 and OR6 genes focus on sex pheromones recognition. OR34 also performed biased expression in male antennae, but till now, the function is unknown. More numbers of ORs were highly expressed in female antennae (Fig. 2), this is also discovered in mosquitos [48]. In *Bombyx mori*, more female biased ORs suggested having function of oviposition cues or male-produced courtship pheromones [49]. This indicated more OR bias in female *C. pinicolalis* might provide more receptors for the detection of correct host plants and sex pheromones as well.

IRs were proven for its multiple functions such as olfaction, chemosensory modalities, taste and response towards non-chemosensory factors like temperature sensing [50–53]. These IRs are highly sensitive to amines and acids [52]. We have identified 20 IRs in *C. pinicolalis* that is much more than the number of IRs reported in *C. punctiferlis*. Indeed, the number of IRs are different in many species. For example, some IRs were exclusively identified in *Spodoptera littoralis* and *Helicoverpa armigera* [54, 55]. Also, many IR genes were identified in gustatory organs in *Drosophila melanogaster* and the long-range attraction to polyamines is mediated by IR76b and IR41a [50, 56]. However, in this study the IR gene family from transcriptome data analyzed only from the *C. pinicolalis* antennae and compared with *C. punctiferlis* antennal dataset. Based on the transcriptome data analysis, we cannot conclude that there are only 20 (*C. pinicolalis*) and 11 (*C. punctiferlis*) [23] IR isoforms in *C. pinicolalis and C. punctiferlis* antenna. The identified IR isoforms in *C. pinicolalis* could help to study gene expansion/deletion and existence of other possible IR isoforms in the *C. punctiferlis* antenna and evolutionary relationship between these two species.

NormFinder and geNorm programs are commonly used to screen and optimize the number of internal reference genes for qRT-PCR analysis [57, 58]. At the same time, the difference between reference genes can be compared, but only one optimal gene can be screened when using the NormFinder [59]. In this research, we used both methods to screen the reference gene. The GeNorm result showed Actin and GAPDH were more stable during different development stages of the *C. pinicolalis*, and NormFinder showed the RP49 as a stable reference gene. This variation may be due to different algorithms coded in this software. Different software were used for calculating the reference gene stability at different developmental stages in the yellow peach moth, RP49 and GAPDH were found to be more stable [60]. Since the expression of the reference gene differs for developmental stages and tissues, therefore the selection of two or more reference genes is useful to calibrate the expression level. Gao et al. [61] reported three different reference genes (Actin, RPL13 and peptidylprolyl isomerase) for different developmental stages in *Aphidius gifuensis*. Also, Actin, GAPDH and RP49 reported being the most stable reference gene in the Calliphoridae family [62]. According to our results, it is recommended to use GAPDH or RP49 at different developmental stages of the *C. pinicolalis*. On another hand, ribosomal proteins are involved in translation and protein synthesis, this recommended us to use RP49 and RPL13 for different tissues in yellow peach moth [60]. Similarly, our findings indicate that both RP49 and RPL13 are the best reference genes for the different body part of the adult.

Furthermore, the female bias genes expression level of OBPs (PBP1, PBP3, PBP4, GOBP1, GOBP2, OBP6, OBP7 and OBP9) were verified by RT-qPCR and extremely consistent with the TMP values obtained from the transcriptome dataset. In addition, the fold change expression results of CSPs, ORs and IRs are consistent with the TMP values (Additional file 2: Figure S2). Therefore, we compared these olfactory-related gene expression levels of *C. pinicolalis* with *C. punctiferalis*, reported by Ge xing et al., 2016 [23]. Gene expression pattern reported from *C. punctiferalis* mostly differs from our study. Noteworthy, most of the ORs (OR2, OR3, OR5, OR6, OR13 and OR15) were significantly expressed in male antenna, whereas in *C. punctiferalis* the ORs were highly expressed in female antenna. At this point, we suggest these ORs might be functionally active in male moths when comparing with *C. punctiferalis* males. On the other hand, OBPs (OBP2, 5 and 6) and GOBPs (GOBP1 and 2) expression patterns were similar to that of *C. punctiferalis*. Exclusively, PBP (PBP1, 2, 3 and 4) genes expression was highly recorded in the *C. punctiferalis* male antenna [23]. In contrast, PBP1, 3 and 4 genes were significantly expressed in *C. pinicolalis* female antenna, only PBP3 had a similar expression pattern. However, most of the gene expression patterns of these olfactory-related proteins were different when compared with *C. punctiferalis* dataset [23], since *C. pinicolalis* is a monophagous pest that mainly feeds on Masson pines.

## Conclusion

We mainly performed a comprehensive analysis of the antennal transcriptome of *C. pinicolalis* and mined many sexual bias expression olfactory related genes. Meanwhile, transcriptome data analysis revealed that most of the olfactory related genes had more than 90% identity with the *C. punctiferlis*. Noteworthy, 4 OBP, 5 OR, 2 IR and 2 CSP genes had 99% sequence similarity with its sibling species *C. punctiferalis*. This study provides a starting point to understand the genetic difference at the molecular level and further intensive studies are required to understand the evolutionary relationship between these two species.

## Methods

### Insects rearing and antennae collection

*C. pinicolalis* larvae were collected from the masson pine in Quanjiao County (32.07 N 117.54 E), Anhui Province, China. Fresh masson pine branch was used to feed the larvae under ambient conditions 27 ± 0.5 °C, with 70–75% relative humidity (RH) and a photo period of 16:8 h light: dark (L:D). After emergence, the moths were feed on 10% honey solution [63]. Three days old moths were selected from both sexes (20 moths/sex) and the antennae were excised for RNA extraction.

### RNA extraction and first-strand cDNA synthesis

Total RNA from male and female antennae was isolated using the Quick-RNA™ MicroPrep Kit (ZYMO Research, USA) according to the manufacturer’s protocol. Ten pairs of antennae were excised from both the sexes. Three biological replicates were maintained (10 pairs/replication). The integrity of the total RNA was analyzed using 1.5% agarose gel electrophoresis [64]. The quality and concentration were analyzed on NanoDrop 2000 spectrophotometer (Thermo Scientific, USA). The cDNA was synthesized by following the instructions from RT™ All-in-One Master Mix Kit (Herogen Biotech, USA). The first strand cDNA synthesis reaction was carried out from 1 μg of total RNA. Anchored oligo (dT) from the kit is used and cDNA was synthesized by following the manufacturer’s protocol. The final cDNA samples were stored at − 20 °C until further analysis.

### Illumina sequencing

Transcriptome sequencing was performed at Novogen Co., Ltd. Beijing, China, and the RNA samples (including 3 biological replicates) were sequenced on the Illumina Hiseq 4000 platform. The raw reads were curated by removing adaptor sequences and low quality reads, then assembled into unigenes using Trinity v2.4.0 [65, 66]. Reads with uncertain nucleotides larger than 10% of the fragment sequence were removed. Trinity de novo program with a default k-mer was used to assemble the clean reads. Sequences redundancy were minimized using CD-HIT program to obtain longest transcript contigs. Annotation-based metrics was adapted for the study. DESeq2 v1.6.3 was used to calculate the identified candidate genes differential expression levels (log2 fold change, *P* < 0.05) [67].

### Unigenes annotation and classification

The unigenes were searched using BLASTX against the non-redundant (nr) NCBI protein database [68]. Using Blast2Go [69], we predicted and classified functions of unigenes by EuKaryotic of orthologous groups (KOG) database [70]. In addition, the online KEGG Automatic Annotation Server (KAAS) was employed for KEGG pathway enrichment analysis following the procedure pathway annotations for unigenes [71, 72].

### Identification of olfactory genes and phylogenetic analyses

The candidate OBPs, ORs and IRs olfactory genes were analyzed using BLASTX, open reading frames (ORFs) were also identified. Phylogenetic tree based on amino acids of these genes was performed with MEGA7.0 software with the neighbour-joining (NJ) method by 1000 replication.

### Analysis of differential gene expression

In order to investigate the expression bias in the antennae of both male and female of *C. pinicolalis* adults, we compared and reported the transcript abundance in units of TPM in both sexes. In the whole dataset of the transcriptome, we identified the interested candidate genes according to their FC, as assessed using corrected *p*-value (*P*) of < 0.05 (*n* = 3). Genes were considered as interesting bias expressed at a FC ≥ 2 and of potential interest if the genes exhibited 1.5 ≤ FC < 2, both with *P* < 0.05.

### Reference genes selection in *C. pinicolalis*

To obtain the stably expressed gene as a reference gene for quantitative real-time PCR (RT-qPCR) and provide a useful message in *C. pinicolalis* study, we selected β-actin gene (Actin), glyceraldehyde 3- GAPDH, RP49 and RPL13 as candidate reference genes based on reference genes in other insect species. The candidate reference gene expression pattern in different development stages (egg, larva, pupa and adult) and the different body part of the adult (antanna, head, throax, abdomen, leg and wings) of the *C. pinicolalis* were assessed by RT-qPCR. Ct values were evaluated by using the GeNorm and NormFinder method to identify the stable reference gene for specific tissues (Additional file 3: Table S2).

### RT-qPCR analysis

The RT-qPCR analysis was performed on select genes to verify the fold changes expression explained in transcriptome data. The primers for RT-qPCR were designed using Primer 3 (http://bioinfo.ut.ee/primer3-0.4.0/primer3/) (Additional file 3: Table S3). The primers efficiency was tested by using 10-fold diluted cDNA samples and the standard curve was generated. The Ct values are plotted against the Log of the cDNA dilutions, efficiency percentage and R^2^ values are within the acceptable range [73]. Quantitative PCR was performed using SybrGreen qPCR Mastermix (DBI Bioscience, Germany), according to manufacturers’ protocol on ABI 7500 Fast (Applied Biosystems, USA) by using the following two-step program: denatured for 2 min at 95 °C followed by 40 cycles: 10 s at 95 °C; 30 s at 60 °C; melting curve analysis was performed from 60 °C to 95 °C to determine the specificity of PCR products. Three independent biological replicates were maintained for all the sample and four technical replicates were performed form each biological sample. The 2^−ΔΔCT^ method was used to calculate relative fold change expression [74]. Fold change expression was analysed using *t*-test, software package SPSS v20.0.

## Supplementary information

**Additional file 1: Figure S1**. A) functional annotation of assembled sequences based on gene ontology (GO) categorization; b) EuKaryotic of orthologous groups (KOG) classification; **c)** is KEGG pathway annotation of the transcriptome. **Figure S2**. Quantitative RT-qPCR expression levels of olfactory genes from female and male moth of *C. pinicolalis*. The expression levels were statistically significant (*t*-test, *P < 0.05, **P < 0.01, ***P < 0.001, NS: not significant). **Figure S3**. Phylogenetic relationship of olfactory-related gene from *C. pinicolalis* and other insects. A: ORs, B: IRs, C: CSPs. Red font represents the genes from *C. pinicolalis*; Cpun, Ofur, Bmor, Cmed, Ehip, Harm and Mcin are the abbreviation of *C. punctiferalis*, *O. furnacalis, B. mori, Cnaphalocrocis medinalis, Eogystia hippophaecolus, Helicoverpa armigera, Macrocentrus cingulum*, respectively.

**Additional file 2: Table S1**. Candidate OBPs, CSPs, ORs and IRs genes in *Conogethes pinicolalis* antennae.

**Additional file 3: Table S2**. Candidate reference genes in *Conogethes pinicolalis* antennae. **Table S3.** Primers for candidate genes by qRT-PCR.

## Data Availability

All data generated or analyzed during this study are included in this published article and its supplementary materials. All Illumina data have been deposited in NCBI’s Sequence Read Archive (SRA) under accession number SRX5250688, SRX5250689, SRX5250690, SRX5250691, SRX5250692 and SRX5250693.

## Abbreviations

Actin: β-actin gene
CSPs: Chemosensory proteins
FC: Differential fold change
GAPDH: Glyceraldehyde 3-phosphate dehydrogenase gene
GO: Gene ontology
GOBPs: General odorant-binding proteins
IRs: Ionotropic receptors
KEGG: Kyoto Encyclopedia of Genes and Genomes
KOG: EuKaryotic of orthologous groups
OBPs: Odorant-binding proteins
ODEs: Odorant degrading enzymes
ORs: Odorant receptors
PBPs: Pheromone-binding proteins
PRs: Pheromones receptors
RP49: Ribosomal protein 49 gene
RPL13: Ribosomal protein L13 gene
RT-qPCR: Quantitative real-time PCR
SNMPs: Sensory neuron membrane proteins
TPM: Transcript per kilobase million
