# Supplementary material for: Sexual-biased gene expression of olfactory-related genes in the antennae of Conogethes pinicolalis (Lepidoptera: Crambidae)
Source: BMC Genomics. 2020 Mar 19;21:244. doi: 10.1186/s12864-020-6648-3 (PMC7081556; doi:10.1186/s12864-020-6648-3)
Supplement: Supplementary file 2 — Additional file 2: Table S1. Candidate OBPs, CSPs, ORs and IRs genes in Conogethes pinicolalis antennae. [file 12864_2020_6648_MOESM2_ESM.docx]

Table S1 Candidate OBPs, CSPs, ORs and IRs genes in Conogethes pinicolalis antennae.

| **Contigs** | **Access No.** | **Gene** | **Residue** | **Full length** | **Top blastx hit** | **Score** | **E-value** | **% ID** | **TPM** | |
| --- | --- | --- | --- | --- | --- | --- | --- | --- | --- | --- |
|  |  |  |  |  |  |  |  |  | **Male** | **Female** |
| Cluster-4268.0 | MK458341 | OBP1 | 402 | No | AHL25274.1\|odorant binding protein 1 [*Macrocentrus cingulum*] | 186 | 2e-58 | 99 | 0.09 | 0.34 |
| Cluster-10503.42126 | MK458342 | OBP2 | 998 | Yes | AHX37224.1\|odorant binding protein 2 [*Conogethes punctiferalis*] | 306 | 3e-102 | 97 | 604.31 | 1198.82 |
| Cluster-10503.40317 | MK458343 | OBP3 | 554 | Yes | AHX37225.1\|odorant binding protein 3 [*Conogethes punctiferalis*] | 210 | 5e-67 | 96 | 0.52 | 1.47 |
| Cluster-10503.31413 | MK458344 | OBP4 | 829 | Yes | ALC76544.1\|odorant binding protein 4 [*Conogethes punctiferalis*] | 278 | 2e-91 | 74 | 0.00 | 0.96 |
| Cluster-10503.43966 | MK458345 | OBP5 | 770 | No | ALC76545.1\|odorant binding protein 5 [*Conogethes punctiferalis*] | 180 | 6e-94 | 99 | 5072.02 | 2287.79 |
| Cluster-10503.41149 | MK458346 | OBP6 | 1158 | Yes | ALC76546.1\|odorant binding protein 6 [*Conogethes punctiferalis*] | 249 | 2e-79 | 96 | 3403.62 | 5210.39 |
| Cluster-10503.32885 | MK458347 | OBP7 | 996 | Yes | ALC76547.1\|odorant binding protein 7 [*Conogethes punctiferalis*] | 288 | 2e-95 | 97 | 135.69 | 314.54 |
| Cluster-10503.45937 | MK458348 | OBP8 | 2078 | Yes | ALC76546.1\|odorant binding protein 8 [*Conogethes punctiferalis*] | 193 | 1e-94 | 95 | 0.98 | 0.60 |
| Cluster-10503.69660 | MK458349 | OBP9 | 873 | Yes | ALC76546.1\|odorant binding protein [*Conogethes punctiferalis*] | 330 | 2e-112 | 98 | 58.19 | 77.25 |
| Cluster-13704.0 | MK458350 | OBP10 | 699 | Yes | APG32532.1\|odorant binding protein [*Conogethes punctiferalis*] | 251 | 1e-82 | 99 | 3.43 | 5.06 |
| Cluster-10503.36927 | MK458351 | OBP11 | 1662 | Yes | APG32533.1\|odorant binding preotein [*Conogethes punctiferalis*] | 280 | 4e-89 | 94 | 5.66 | 2.70 |
| Cluster-10503.41998 | MK458352 | OBP12 | 1772 | Yes | APG32534.1\|odorant binding preotein [*Conogethes punctiferalis*] | 221 | 2e-50 | 98 | 3.79 | 5.39 |
| Cluster-10503.41821 | MK458353 | OBP13 | 257 | No | APG32535.1\|odorant binding preotein [*Conogethes punctiferalis*] | 124 | 1e-34 | 88 | 1609.77 | 758.88 |
| Cluster-10503.30173 | MK458354 | OBP14 | 712 | No | APG32537.1\|odorant binding preotein [*Conogethes punctiferalis*] | 271 | 2e-34 | 95 | 21.87 | 16.22 |
| Cluster-10503.73845 | MK458355 | OBP15 | 1225 | Yes | APG32538.1\|odorant binding preotein [*Conogethes punctiferalis*] | 297 | 1e-97 | 98 | 0.13 | 0.08 |
| Cluster-10503.41510 | MK458356 | OBP16 | 693 | Yes | APG32540.1\|odorant binding preotein [*Conogethes punctiferalis*] | 226 | 3e-72 | 97 | 4416.13 | 4719.04 |
| Cluster-10503.41083 | MK458357 | OBP17 | 623 | Yes | APG32541.1\|odorant binding preotein [*Conogethes punctiferalis*] | 307 | 3e-104 | 98 | 409.94 | 316.07 |
| Cluster-10503.19709 | MK458358 | OBP18 | 2208 | Yes | APG32542.1\|odorant binding preotein [*Conogethes punctiferalis*] | 353 | 5e-115 | 99 | 2.10 | 2.54 |
| Cluster-10503.41671 | MK458359 | OBP19 | 585 | Yes | APG32543.1\|odorant binding preotein [*Conogethes punctiferalis*] | 252 | 1e-82 | 97 | 744.65 | 951.89 |
| Cluster-10503.36898 | MK458360 | OBP20 | 1462 | Yes | ALT31650.1\|odorant-binding protein 20 [Cnaphalocrocis medinalis] | 261 | 9e-83 | 95 | 368.99 | 398.05 |
| Cluster-10503.41407 | MK458335 | GOBP1 | 610 | Yes | APG32536.1\|odorant binding preotein [*Conogethes punctiferalis*] | 297 | 3e-100 | 95 | 3503.74 | 6305.09 |
| Cluster-10503.41481 | MK458336 | GOBP2 | 898 | Yes | AMY16433.1\|general odorant binding protein 2 [*Conogethes punctiferalis*] | 191 | 4e-57 | 99 | 4996.65 | 9316.62 |
| Cluster-10503.39766 | MK458337 | PBP1 | 588 | Yes | AVL25107.1\|PBP1 [*Conogethes punctiferalis*] | 192 | 2e-59 | 97 | 340.87 | 970.18 |
| Cluster-10503.38321 | MK458338 | PBP2 | 733 | Yes | ALC76550.1\|pheromone binding protein 2 [*Conogethes punctiferalis*] | 190 | 5e-33 | 95 | 320.11 | 21.55 |
| Cluster-10503.42276 | MK458339 | PBP3 | 1807 | Yes | ALC76551.1\|pheromone binding protein 3 [*Conogethes punctiferalis*] | 338 | 1e-100 | 95 | 671.14 | 1699.22 |
| Cluster-10503.41761 | MK458340 | PBP4 | 2058 | Yes | ALC76549.1\|pheromone binding protein 5 [*Conogethes punctiferalis*] | 329 | 3e-106 | 93 | 328.03 | 3346.67 |
| Cluster-10503.79683 | MK458361 | OR1 | 1548 | No | ARO76407.1\|odorant receptor 1 [*Conogethes punctiferalis*] | 890 | 0 | 95 | 5.58 | 0.00 |
| Cluster-10503.39165 | MK458362 | OR2 (Orco) | 2305 | Yes | ARO76408.1\|odorant receptor 2 [*Conogethes punctiferalis*] | 952 | 0 | 99 | 526.17 | 398.63 |
| Cluster-10503.3804 | MK458363 | OR3 | 1472 | Yes | ARO76409.1\|odorant receptor 3 [*Conogethes punctiferalis*] | 641 | 0 | 94 | 25.08 | 0.00 |
| Cluster-10503.5472 | MK458364 | OR4 | 3148 | No | ARO76410.1\|odorant receptor 4 [*Conogethes punctiferalis*] | 868 | 0 | 92 | 0.38 | 0.17 |
| Cluster-10503.5037 | MK458365 | OR5 | 1776 | Yes | ARO76411.1\|odorant receptor 5 [*Conogethes punctiferalis*] | 758 | 0 | 95 | 4.02 | 3.02 |
| Cluster-10503.3438 | MK458366 | OR6 | 1499 | Yes | ARO76412.1\|odorant receptor 6 [*Conogethes punctiferalis*] | 805 | 0 | 95 | 134.97 | 0.09 |
| Cluster-10503.639 | MK458367 | OR7 | 1564 | Yes | ARO76413.1\|odorant receptor 7 [*Conogethes punctiferalis*] | 555 | 0 | 90 | 0.13 | 0.00 |
| Cluster-10503.18492 | MK458368 | OR8 | 1190 | No | ARO76414.1\|odorant receptor 8 [*Conogethes punctiferalis*] | 339 | 3e-110 | 77 | 0.19 | 0.23 |
| Cluster-10503.20828 Cluster-10503.15452 | MK458369 | OR10 | 1760 | Yes | ARO76416.1\|odorant receptor 10 [*Conogethes punctiferalis*] | 656 | 5e-165 | 87 | 0.64 | 1.18 |
| Cluster-10503.25758 | MK458370 | OR11 | 1382 | Yes | ARO76417.1\|odorant receptor 11 [*Conogethes punctiferalis*] | 683 | 0 | 97 | 0.39 | 0.23 |
| Cluster-10503.23000 | MK458371 | OR12 | 8179 | No | ARO76418.1\|odorant receptor 12 [*Conogethes punctiferalis*] | 664 | 0 | 93 | 7.20 | 11.53 |
| Cluster-10503.49596 | MK458372 | OR13 | 1300 | Yes | ARO76419.1\|odorant receptor 13 [*Conogethes punctiferalis*] | 752 | 0 | 96 | 10.59 | 14.61 |
| Cluster-10503.14972 | MK458373 | OR14 | 1572 | No | ARO76420.1\|odorant receptor 14 [*Conogethes punctiferalis*] | 798 | 0 | 97 | 1.55 | 3.26 |
| Cluster-10503.73785 | MK458374 | OR15 | 2419 | Yes | ARO76421.1\|odorant receptor 15 [*Conogethes punctiferalis*] | 673 | 0 | 90 | 10.55 | 15.73 |
| Cluster-10503.29481 | MK458375 | OR16 | 1887 | Yes | ARO76422.1\|odorant receptor 16 [*Conogethes punctiferalis*] | 794 | 0 | 91 | 6.30 | 9.92 |
| Cluster-10503.66784 | MK458376 | OR17 | 7580 | No | ARO76423.1\|odorant receptor 17 [*Conogethes punctiferalis*] | 790 | 0 | 98 | 0.00 | 0.01 |
| Cluster-10503.70820 | MK458377 | OR18 | 2028 | No | ARO76424.1\|odorant receptor 18 [*Conogethes punctiferalis*] | 786 | 0 | 95 | 1.62 | 1.38 |
| Cluster-10503.30701 | MK458378 | OR19 | 1674 | Yes | ARO76425.1\|odorant receptor 19 [*Conogethes punctiferalis*] | 726 | 0 | 89 | 3.03 | 5.01 |
| Cluster-10503.53919 Cluster-10503.53918 | MK458379 | OR20 | 1134 | Yes | ARO76426.1\|odorant receptor 20 [*Conogethes punctiferalis*] | 729 | 0 | 96 | 0.46 | 1.47 |
| Cluster-10503.25689 Cluster-10503.23609 | MK458380 | OR21 | 3462 | Yes | BAR43463.1\|putative olfactory receptor 21, partial [Conogethes punctiferalis] | 536 | 3e-178 | 77 | 9.99 | 12.35 |
| Cluster-10503.66444 | MK458381 | OR22 | 2824 | No | BAR43464.1\|putative olfactory receptor 22 [*Ostrinia furnacalis*] | 535 | 9e-180 | 74 | 2.43 | 3.96 |
| Cluster-10503.36169 | MK458382 | OR23 | 1741 | Yes | ARO76428.1\|odorant receptor 23 [*Conogethes punctiferalis*] | 730 | 0 | 93 | 7.40 | 10.56 |
| Cluster-10503.67479 | MK458383 | OR24 | 2296 | Yes | ARO76429.1\|odorant receptor 24 [*Conogethes punctiferalis*] | 897 | 0 | 98 | 4.43 | 17.48 |
| Cluster-10503.12749 | MK458384 | OR25 | 2027 | Yes | ARO76430.1\|odorant receptor 25 [*Conogethes punctiferalis*] | 833 | 0 | 93 | 0.27 | 0.24 |
| Cluster-10503.69718 | MK458385 | OR26 | 1465 | Yes | BAR43468.1\|putative olfactory receptor 26 [*Ostrinia furnacalis*] | 491 | 7e-169 | 65 | 7.46 | 10.39 |
| Cluster-10503.28230 | MK458386 | OR27 | 1563 | Yes | ARO76432.1\|odorant receptor 27 [*Conogethes punctiferalis*] | 740 | 0 | 99 | 11.67 | 17.48 |
| Cluster-10503.30271 | MK458387 | OR28 | 1110 | Yes | ARO76433.1\|odorant receptor 28 [*Conogethes punctiferalis*] | 586 | 0 | 94 | 0.43 | 0.26 |
| Cluster-10503.70890 | MK458388 | OR29 | 1540 | Yes | ARO76434.1\|odorant receptor 29 [*Conogethes punctiferalis*] | 734 | 0 | 88 | 0.41 | 2.00 |
| Cluster-10503.34687 | MK458389 | OR30 | 1560 | Yes | BAR43472.1\|putative olfactory receptor 30 [*Ostrinia furnacalis*] | 573 | 0 | 69 | 3.43 | 4.83 |
| Cluster-10503.59568 | MK458390 | OR31 | 1066 | Yes | ARO76436.1\|odorant receptor 31 [*Conogethes punctiferalis*] | 564 | 0 | 83 | 7.71 | 14.35 |
| Cluster-10503.32740 | MK458391 | OR32 | 1411 | Yes | ARO76437.1\|odorant receptor 32 [*Conogethes punctiferalis*] | 712 | 0 | 93 | 1.18 | 1.89 |
| Cluster-10503.10324 | MK458392 | OR33 | 1401 | No | ARO76438.1\|odorant receptor 33 [*Conogethes punctiferalis*] | 774 | 0 | 99 | 2.71 | 3.69 |
| Cluster-10503.73859 | MK458393 | OR34 | 1159 | No | ARO76439.1\|odorant receptor 34 [*Conogethes punctiferalis*] | 444 | 1e-153 | 63 | 8.55 | 0.06 |
| Cluster-10503.69056 | MK458394 | OR35 | 1957 | Yes | ARO76440.1\|odorant receptor 35 [*Conogethes punctiferalis*] | 882 | 0 | 99 | 6.81 | 10.67 |
| Cluster-10503.10992 | MK458395 | OR36 | 1093 | Yes | ARO76441.1\|odorant receptor 36 [*Conogethes punctiferalis*] | 409 | 3e-98 | 93 | 0.57 | 0.07 |
| Cluster-10503.9096 | MK458396 | OR37 | 2214 | Yes | ARO76442.1\|odorant receptor 37 [*Conogethes punctiferalis*] | 735 | 0 | 96 | 5.09 | 7.80 |
| Cluster-10503.16600 | MK458397 | OR38 | 1428 | Yes | ARO76443.1\|odorant receptor 38 [*Conogethes punctiferalis*] | 657 | 0 | 78 | 6.11 | 7.17 |
| Cluster-10503.58595 | MK458398 | OR39 | 1505 | Yes | BAR43478.1\|putative olfactory receptor 36 [*Ostrinia furnacalis*] | 554 | 0 | 79 | 2.79 | 4.30 |
| Cluster-10503.66339 | MK458399 | OR40 | 1543 | Yes | ARO76445.1\|olfactory receptor 40 [Cnaphalocrocis medinalis] | 743 | 0 | 91 | 3.22 | 6.18 |
| Cluster-10503.76047 | MK458400 | OR41 | 1789 | Yes | ARO76446.1\|odorant receptor 41 [*Conogethes punctiferalis*] | 644 | 0 | 93 | 0.14 | 0.27 |
| Cluster-10503.74009 | MK458401 | OR42 | 1668 | No | ARO76447.1\|odorant receptor 42 [*Conogethes punctiferalis*] | 686 | 0 | 92 | 1.05 | 3.51 |
| Cluster-10503.11496 | MK458402 | OR43 | 1369 | No | ARO76448.1\|odorant receptor 43 [*Conogethes punctiferalis*] | 581 | 0 | 96 | 10.10 | 12.37 |
| Cluster-10503.45661 | MK458403 | OR44 | 1955 | No | ARO76449.1\|odorant receptor 44 [*Conogethes punctiferalis*] | 684 | 0 | 86 | 2.80 | 3.52 |
| Cluster-10503.8969 | MK458404 | OR45 | 1553 | Yes | ARO76450.1\|odorant receptor 45 [*Conogethes punctiferalis*] | 508 | 3e-175 | 63 | 5.82 | 9.77 |
| Cluster-10503.29246 | MK458405 | OR46 | 2272 | Yes | ANZ03153.1\|olfactory receptor 40 [*Cnaphalocrocis medinalis*] | 707 | 0 | 78 | 4.56 | 7.93 |
| Cluster-9485.0 | MK458406 | OR47 | 716 | No | ARO76452.1\|odorant receptor 47 [*Conogethes punctiferalis*] | 299 | 4e-100 | 99 | 0.32 | 0.43 |
| Cluster-10503.55928 Cluster-10503.55927 | MK458407 | OR48 | 1599 | Yes | ARO76453.1\|odorant receptor 48 [*Conogethes punctiferalis*] | 437 | 1e-148 | 79 | 0.00 | 8.41 |
| Cluster-10503.22889 Cluster-10503.63090 | MK458408 | OR49 | 1219 | Yes | ARO76454.1\|odorant receptor 49 [*Conogethes punctiferalis*] | 114 | 3e-23 | 91 | 3.94 | 5.81 |
| Cluster-10503.24940 | MK458409 | OR50 | 1532 | Yes | ARO76455.1\|odorant receptor 50 [*Conogethes punctiferalis*] | 800 | 0 | 94 | 2.11 | 2.17 |
| Cluster-10503.63760 Cluster-10503.70828 | MK458410 | OR51 | 1364 | No | ARO76456.1\|odorant receptor 51 [*Conogethes punctiferalis*] | 647 | 0 | 93 | 1.82 | 3.50 |
| Cluster-10503.73834 | MK458411 | OR52 | 1852 | No | ARO76457.1\|odorant receptor 52 [*Conogethes punctiferalis*] | 728 | 0 | 91 | 0.87 | 0.77 |
| Cluster-10503.23967 | MK458412 | OR53 | 4285 | Yes | ARO76458.1\|odorant receptor 53 [*Conogethes punctiferalis*] | 691 | 0 | 90 | 0.44 | 2.36 |
| Cluster-10503.66442 | MK458413 | OR54 | 3272 | Yes | ARO76459.1\|odorant receptor 54 [*Conogethes punctiferalis*] | 853 | 0 | 92 | 0.00 | 6.02 |
| Cluster-10503.51937 | MK458414 | OR55 | 2337 | Yes | ARO76460.1\|odorant receptor 55 [*Conogethes punctiferalis*] | 839 | 0 | 94 | 2.18 | 6.42 |
| Cluster-10503.13642 | MK458415 | OR56 | 1668 | Yes | ARO76461.1\|odorant receptor 56 [*Conogethes punctiferalis*] | 690 | 0 | 89 | 0.31 | 0.55 |
| Cluster-10503.17349 | MK458416 | IR1 | 997 | No | BAR64812.1\|ionotropic receptor [*Ostrinia furnacalis*] | 444 | 2e-152 | 78 | 1.89 | 2.28 |
| Cluster-10503.45381 | MK458417 | IR2 | 682 | No | AIG51915.1\|ionotropic receptor, partial [*Helicoverpa armigera*] | 118 | 4e-28 | 32 | 0.38 | 0.06 |
| Cluster-10503.11677 Cluster-10503.11204 | MK458418 | IR3 | 4729 | Yes | ARO76466.1\|ionotropic receptor 3 [*Conogethes punctiferalis*] | 1299 | 0 | 99 | 4.41 | 4.70 |
| Cluster-10503.46676 | MK458419 | IR4 | 2390 | Yes | ARO76467.1\|ionotropic receptor 4 [*Conogethes punctiferalis*] | 1057 | 0 | 98 | 40.85 | 62.25 |
| Cluster-10503.45198 | MK458420 | IR5 | 3877 | No | ARO76468.1\|ionotropic receptor 5 [*Conogethes punctiferalis*] | 1484 | 0 | 81 | 14.39 | 20.19 |
| Cluster-10503.63470 | MK458421 | IR6 | 2885 | Yes | ARO76469.1\|ionotropic receptor 6 [*Conogethes punctiferalis*] | 1348 | 0 | 81 | 10.85 | 13.09 |
| Cluster-10503.74785 | MK458422 | IR7 | 2786 | No | ARO76470.1\|ionotropic receptor 7 [*Conogethes punctiferalis*] | 1089 | 0 | 97 | 0.42 | 0.22 |
| Cluster-10503.28593 | MK458424 | IR25a | 4948 | No | ARO76463.1\|ionotropic receptor 25a [*Conogethes punctiferalis*] | 1797 | 0 | 99 | 0.12 | 0.13 |
| Cluster-10503.20615 | MK458425 | IR31a | 2189 | No | AOG12849.1\|ionotropic receptor [*Eogystia hippophaecolus*] | 617 | 0 | 52 | 7.29 | 5.97 |
| Cluster-10503.14097 | MK458426 | IR40a | 2330 | Yes | APY22697.1\|ionotropic receptor IR40a [*Cnaphalocrocis medinalis*] | 1220 | 0 | 90 | 1.10 | 1.44 |
| Cluster-10503.39972 | MK458427 | IR64a | 4123 | Yes | APY22699.1\|ionotropic receptor IR64a [*Cnaphalocrocis medinalis*] | 830 | 0 | 70 | 5.89 | 3.62 |
| Cluster-10503.17469 | MK458428 | IR75d | 1802 | No | ARO70544.1\|antennal ionotropic receptor 75d-2 [*Dendrolimus punctatus*] | 623 | 0 | 67 | 6.83 | 9.63 |
| Cluster-10503.20733 Cluster-10503.10927 | MK458429 | IR75p | 1235 | Yes | ALT31628.1\|ionotropic receptor 75p [*Cnaphalocrocis medinalis*] | 467 | 5e-159 | 75 | 3.01 | 1.62 |
| Cluster-10503.53579 | MK458430 | IR75p1 | 3455 | Yes | BAR64805.1\|ionotropic receptor [*Ostrinia furnacalis*] | 867 | 0 | 69 | 5.59 | 7.03 |
| Cluster-10503.20731 | MK458431 | IR75p2 | 1355 | No | BAR64806.1\|ionotropic receptor [*Ostrinia furnacalis*] | 562 | 0 | 64 | 1.92 | 1.09 |
| Cluster-11206.0 | MK458432 | IR75p3 | 615 | No | BAR64807.1\|ionotropic receptor [*Ostrinia furnacalis*] | 267 | 7e-83 | 88 | 0.23 | 0.26 |
| Cluster-10503.27492 | MK458423 | IR75q1 | 592 | No | ARO76471.1\|ionotropic receptor 8 [*Conogethes punctiferalis*] | 166 | 2e-46 | 95 | 1.09 | 1.58 |
| Cluster-10503.31133 | MK458433 | IR75q2 | 5577 | No | BAR64808.1\|ionotropic receptor [*Ostrinia furnacalis*] | 1149 | 0 | 78 | 2.45 | 4.21 |
| Cluster-10503.54328 | MK458434 | IR93a | 2721 | Yes | ALT31632.1\|ionotropic receptor 93a [*Cnaphalocrocis medinalis*] | 1453 | 0 | 96 | 14.08 | 14.70 |
| Cluster-10503.43571 | MK574125 | CSP1 | 1280 | No | AHX37218.1\|chemosensory protein 1 [*Conogethes punctiferalis*] | 154 | 1e-41 | 96 | 1033.67 | 1002.99 |
| Cluster-10503.41197 | MK574126 | CSP2 | 2865 | Yes | AHX37219.1\|chemosensory protein 2 [*Conogethes punctiferalis*] | 259 | 8e-78 | 96 | 451.04 | 554.18 |
| Cluster-10503.56691 | MK574127 | CSP3 | 384 | Yes | APG32545.1\|chemosensory protein 3 [*Conogethes punctiferalis*] | 191 | 1e-60 | 90 | 57.61 | 59.45 |
| Cluster-10503.40769 | MK574128 | CSP4 | 1448 | Yes | AHX37226.1\|chemosensory protein 4 [*Conogethes punctiferalis*] | 226 | 5e-69 | 96 | 1147.73 | 1690.67 |
| Cluster-10503.62040 | MK574129 | CSP5 | 1083 | Yes | AHX37227.1\|chemosensory protein 5 [*Conogethes punctiferalis*] | 246 | 1e-78 | 98 | 4.59 | 12.15 |
| Cluster-10503.40078 | MK574130 | CSP6 | 603 | Yes | AHX37220.1\|chemosensory protein 6 [*Conogethes punctiferalis*] | 228 | 1e-67 | 97 | 2538.19 | 2531.75 |
| Cluster-10503.42171 | MK574131 | CSP7 | 1350 | Yes | AHX37221.1\|chemosensory protein 7 [*Conogethes punctiferalis*] | 201 | 1e-59 | 97 | 1205.59 | 1307.27 |
| Cluster-10503.36219 | MK574132 | CSP8 | 392 | No | AHX37222.1\|chemosensory protein 8 [*Conogethes punctiferalis*] | 172 | 3e-53 | 99 | 1.19 | 2.41 |
| Cluster-10503.2076 | MK574133 | CSP9 | 832 | Yes | APG32546.1\|chemosensory protein [*Conogethes punctiferalis*] | 241 | 5e-78 | 96 | 0.76 | 0.47 |
| Cluster-10503.76346 | MK574134 | CSP10 | 1176 | Yes | APG32546.2\|chemosensory protein [*Conogethes punctiferalis*] | 197 | 8e-71 | 99 | 9.02 | 9.62 |
| Cluster-10503.41605 | MK574135 | CSP11 | 578 | Yes | APG32548.1\|chemosensory protein [*Conogethes punctiferalis*] | 219 | 2e-59 | 96 | 791.96 | 1957.60 |
| Cluster-10503.1092 | MK574136 | CSP12 | 419 | Yes | AIX97831.1\|chemosensory protein [*Cnaphalocrocis medinalis*] | 176 | 1e-54 | 79 | 0.76 | 1.05 |
| Cluster-10503.71506 | MK574137 | CSP13 | 615 | Yes | APG32550.1\|chemosensory protein [*Conogethes punctiferalis*] | 206 | 2e-64 | 88 | 28.85 | 16.17 |
| Cluster-10503.41355 | MK574138 | CSP14 | 1110 | Yes | APG32551.1\|chemosensory protein [*Conogethes punctiferalis*] | 228 | 3e-71 | 92 | 574.50 | 1272.96 |
| Cluster-10503.42061 | MK574139 | CSP15 | 879 | Yes | APG32552.1\|chemosensory protein [*Conogethes punctiferalis*] | 237 | 4e-76 | 94 | 0.59 | 0.40 |
| Cluster-10503.2507 | MK574140 | CSP16 | 922 | No | BAV56820.1\|chemosensory protein 16 [*Ostrinia furnacalis*] | 156 | 3e-44 | 68 | 0.52 | 0.63 |
| Cluster-10503.41781 | MK574141 | CSP17 | 381 | Yes | AIX97836.1\|chemosensory protein [*Cnaphalocrocis medinalis*] | 199 | 9e-62 | 65 | 599.71 | 2706.05 |
| Cluster-10503.72536 | MK574142 | CSP18 | 1181 | Yes | AIX97829.1\|chemosensory protein [*Cnaphalocrocis medinalis*] | 204 | 1e-61 | 80 | 1.05 | 1.56 |
| Cluster-10503.64572 | MK574143 | CSP19 | 1078 | No | AIX97828.1\|chemosensory protein [*Cnaphalocrocis medinalis*] | 154 | 5e-43 | 85 | 8.89 | 10.61 |
