# Supplementary material for: Sexual-biased gene expression of olfactory-related genes in the antennae of Conogethes pinicolalis (Lepidoptera: Crambidae)
Source: BMC Genomics. 2020 Mar 19;21:244. doi: 10.1186/s12864-020-6648-3 (PMC7081556; doi:10.1186/s12864-020-6648-3)
Supplement: Supplementary file 3 — Additional file 3: Table S2. Candidate reference genes in Conogethes pinicolalis antennae. Table S3. Primers for candidate genes by qRT-PCR. [file 12864_2020_6648_MOESM3_ESM.docx]

Table S2 Candidate reference genes in Conogethes *pinicolalis* antennae

| **Contigs** | **Access No.** | **Gene** | **Residue** | **Full length** | **Top blastx hit** | **Score** | **E-value** | **% ID** |
| --- | --- | --- | --- | --- | --- | --- | --- | --- |
|  |  |  |  |  |  |  |  |  |
| Cluster-10503.45313 | MK458333 | Actin | 2397 | Yes | WP_110883488.1\|actin, cytoplasmic 2 [*Microbacterium arborescens*] | 788 | 0 | 100 |
| Cluster-10503.42932 | MK458330 | RP49 | 490 | Yes | ASN77689.1\|ribosomal protein 49 [*Conogethes punctiferalis*] | 276 | 2e-93 | 100 |
| Cluster-10503.37951 | MK458334 | GAPDH | 1335 | Yes | ASN77688.1\|glyceraldehyde-3-phosphate dehydrogenase [*Conogethes punctiferalis*] | 644 | 0 | 100 |
| Cluster-10503.38723 | MK458331 | RPL13 | 661 | Yes | ASN77691.1\|ribosomal protein L13 [*Conogethes punctiferalis*] | 390 | 1e-136 | 100 |

Table S3 Primers for candidate genes by qRT-PCR.

| Primer Name | Sequence (5'-3') |
| --- | --- |
| OP2-F | GACAGATGAACAAAGAGCC |
| OP2-R | AAAAAGCAGGAAGCAGAAT |
| OP6-F | TGAAGTGCTATATGTTTTGCCT |
| OP6-R | ATCATCTTAGTTGTCCTGTCCG |
| OP7-F | CAACAAAATAGTTCACGA |
| OP7-R | CAAATACAAAGTTCTCCG |
| OP8-F | GATGAAGAACGGAAAACC |
| OP8-R | ACGGAAAGAAGAAAATGC |
| OP9-F | ATGTTCCGCGCTCATTCG |
| OP9R | CCTGGTCGCCATCTTGCT |
| OP16-F | TGGAACACAGCGAGAAAT |
| OP16-R | AGCAGGACAGAAGCAACT |
| OP19-F | AGAAGGCGTAGAAAAACC |
| OP19-R | CTTGGAACAATCGGGAGG |
| GOBP1-F | GCACCAGGAGTTCAAGTTCG |
| GOBP1-R | ACCTCCTCGAACTGCTTCTC |
| GOBP2-F | TTACTGCAGGAGGACACCAG |
| GOBP2-R | TGGTAATCTCCGGAGCGATG |
| PBP1-F | GATTACCAGCTGACGAACCG |
| PBP1-R | AGTTGTCGTCCATTTTGCCC |
| PBP3-F | GACTCCGGTTGCATGTTCAG |
| PBP3-R | ATCTCCAGCATTCGGTCACA |
| PBP4-F | TAGCCGACGACATGATCCAA |
| PBP4-R | AAGCGTTCTCATGGTGCATC |
| OP5-F | TGATGTCTGACGATGGTGT |
| OP5-R | TTATCTTTATGCTTGCGGT |
| OP13-F | GGAAGAGAAACGAGAAAAA |
| OP13-R | GTGCCGCACGACCGGAAAT |
| OP14-F | CTTCATCGCTTGCGTCTTCA |
| OP14-R | CAGGGGTTTCGTCGTTCACT |
| OP17-F | GCTTTATTAGCCCTCTCCAT |
| OP17-R | AGTTTACCCTTCACATCGTC |
| OP20-F | ATTGTGTTCGCTGTCTGCCT |
| OP20-R | TTGTTGACCAACTGCTCTTC |
| PBP2-F | CATAAAGGGAAAACGGCGGA |
| PBP2-R | TTCCAGCAAGACCTCAGTGT |
| OR13-F | CGACCTGAATCTTCTGCTC |
| OR13-R | AATCTATGATACCTTGCCA |
| OR15-F | TATTTACAGCACCGATTG |
| OR15-R | GGAGAGAGGGAACATTTT |
| OR24-F | TGACCAGTTCATTCCTTTTC |
| OR24-R | GTACTTCCACGGTATCCATA |
| OR27-F | AGATAGGAATGTTGTGTTGG |
| OR27-R | CTGGGCTCTCTGGATAAGGA |
| OR31-F | GCTTTTCACCAACTTGTCTC |
| OR31-R | CTCTTAATAATCTCCTTCCC |
| OR43-F | TAGCACCTTGTCTAGGCATC |
| OR43-R | AACCACTTCTCTCTTTTCGG |
| OR2-F | CATTTGTGGGGTTACTGTCT |
| OR2-R | AGTTTCATTGGTTTCCTTGT |
| OR3-F | TATAGCGTACAGTGGGAGTG |
| OR3-R | AAATGTTGTTTTCAAAATCG |
| OR5-F | ATAATAACAGAGAAGAAAAT |
| OR5-R | ATAAGTAAATCAAGGCAACA |
| OR6-F | GCTGTCTTTGTCTGTCTTGG |
| OR6-R | ATCTGGTTCACCTTCTCGTA |
| IR4-F | AAATGGGTCACTTATTGGCA |
| IR4-R | TAATGAATCCTGTGGGTTGT |
| IR5-F | TGAAAACATACAGGCCACAG |
| IR5-R | TCACCATCCCAATAAAACAA |
| IR6-F | TCTCTGACAAACACACCCTT |
| IR6-R | TGAAAATCCAATACCAACCT |
| IR93-F | GGTGGTAATGATGTGTTGTGT |
| IR93-R | AGGAAGTCTTTTTTAAGGCTG |

F is forward primer and R is reverse primer.
